# Bidirectional gut-brain-microbiota axis as a potential link between inflammatory bowel disease and ischemic stroke

**DOI:** 10.1186/s12974-018-1382-3

**Published:** 2018-12-11

**Authors:** Liang Zhao, Qiutang Xiong, Creed M. Stary, Omer Kamal Mahgoub, Yingze Ye, Lijuan Gu, Xiaoxing Xiong, Shengmei Zhu

**Affiliations:** 1 Department of Gastroenterology, Renmin Hospital of Wuhan University, Wuhan, China; 2 Diabetes Research Center, Renmin Hospital of Wuhan University, Wuhan, China; 3 Department of Anesthesiology, Perioperative and Pain Medicine, Stanford University School of Medicine, Stanford, CA 94305 USA; 4 Central Laboratory, Renmin Hospital of Wuhan University, Wuhan, China; 5 Department of Anesthesiology, The First Affiliated Hospital, College of Medicine, Zhejiang University, Hangzhou, 310000 Zhejiang China; 6 Department of Neurosurgery, Renmin Hospital of Wuhan University, 99 Zhang Zhidong Rd, Wuhan, 430060 Hubei China

**Keywords:** Gut-brain-microbiota axis, Inflammatory, Inflammatory bowel disease, Adrenocorticotropic hormone, Ischemic stroke

## Abstract

Emerging evidence suggests that gut-brain-microbiota axis (GBMAx) may play a pivotal role linking gastrointestinal and neuronal disease. In this review, we summarize the latest advances in studies of GBMAx in inflammatory bowel disease (IBD) and ischemic stroke. A more thorough understanding of the GBMAx could advance our knowledge about the pathophysiology of IBD and ischemic stroke and help to identify novel therapeutic targets via modulation of the GBMAx.

## Introduction

There exist a bidirectional communication and interaction between the gut and brain [1]. The structure and function of the brain can be modulated by the gut, and conversely, the brain regulates the gut microenvironment and microbiota composition. Emerging evidence indicates that the gut-brain interaction is significantly modulated by microbiota, which acts as a relatively independent and variable component [2]. Therefore the gut-brain-microbiota axis (GBMAx) has been recently been described to underscore the contribution of microbiota in the bidirectional communication of the gut and brain [3]. In fact, dysregulation of the GBMAx has been implicated in a variety of gastrointestinal and central nervous system (CNS) diseases. A better understanding of the gut-brain-microbiota axis interactions will advance our knowledge about the etiology of those diseases and allow novel therapeutic targets to be discovered.

Inflammatory bowel disease (IBD) is a gut disorder which is characterized by a recurrent and chronic gastrointestinal inflammation. Recent evidence suggests that chronic inflammation in IBD may result from an aberrant immune response towards the abnormal gut microbiota in genetically susceptible individuals [4]. Notably, patients with IBD have a higher risk of cerebrovascular thromboembolism, which is the most grievous complication of the central nervous system (CNS), than the non-IBD population [5]. The mechanism of the high risk of ischemic stroke in IBD patients remains elusive, and the significance of such connection remains largely underestimated in clinical practice [2]. In this review, we will present an overview of the latest advances on the GBMAx in the interaction between inflammatory bowel disease and ischemic stroke. A comprehensive understanding of the GBMAx is critically important to identify novel therapeutic options for gastrointestinal and neurological disorders both collectively and independently.

## The gut-brain-microbiota axis

**Fig. 1:**
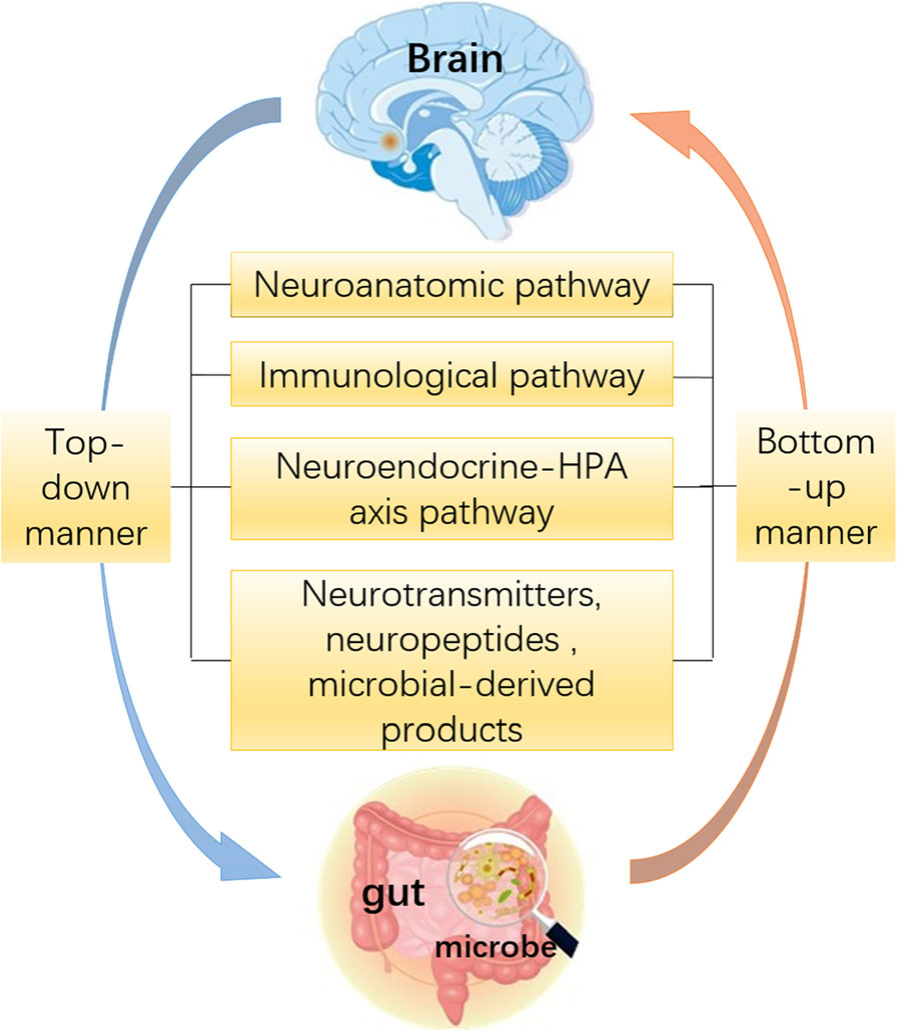
General concept of bidirectional gut-brain-microbiota axis (GBMAx). The brain regulates the gut and its microbiota via neuroanatomic, immunological, and neuroendocrine-HPA axis pathways, communicating via neurotransmitters, neuropeptides, or microbial-derived products effecting gut microbiota. Accordingly, the gut microbiota influences the brain. These two manners form the bidirectional communication and interactions between the gut and brain

The gut-brain-microbiota axis consists of the following essential components: the central nervous system (CNS); the autonomic nervous system; the enteric nervous system (ENS); neurotransmitters, hormone and neuropeptides; the hypothalamic-pituitary-adrenal axis (HPA); intestinal microenvironment (the intestinal barrier, gut microbiota, and their metabolic products, entero-endocrine, and immune system), and the blood-brain barrier [2]. The interactions on GBMAx are mediated via several neuro-immune-endocrine pathways, schematically outlined in Fig. 1.

### Neuroanatomic pathways

There are two neuroanatomic pathways for the bidirectional communication of GBMAx. One is the direct gut-brain communication via both of the vagus nerve (VN) and autonomic nervous system (ANS) in the spinal cord. The other is communication between the enteric nervous system (ENS) in the gut and ANS as well as the VN within the spinal cord [6]. The visceral signals produced in gastrointestinal lumen and mucosa include luminal osmolarity, carbohydrate levels, mucosal mechanical distortion, cytostatic drugs, bacterial products, and visceral pain. Those signals are processed and integrated by various ANS centers and feedback loops in the CNS and spinal cord. The core components involved in the process are listed as follows: (1) enteric neural networks; (2) visceral reflex loop modulated by prevertebral ganglia; (3) ANS centers in the spinal cord (sympathetic nerves at T5-L2 level, parasympathetic ones at S2-S4 level), the brainstem nucleus tractus solitarius, and the dorsal motor nucleus of vagal afferent nerve fibers; and (4) the advanced interconnected brain centers including the basal ganglia and brainstem nuclei spreading to the thalamus, insular vortex, and lobus limbicus [2]. In response to the signals originated from the gut, the CNS sends regulatory information to the intestinal microenvironment via the ENS, neuronal-glial-epithelial unit, or directly acts on gastrointestinal effector cells through the ANS and the neuroendocrine system to regulate the contraction of smooth muscles and activity of glands and blood vessels [2].

The significance of crosstalk between the gut microbiota and the CNS in the regulation of behavior has been increasingly recognized. It has been noted that gut microbiota can regulate neuronal activities by stimulating the ENS and afferent signaling via VN from the gut. Using an animal model of chronic colitis, it was demonstrated that an anxiety-like behavior was a result of a disrupted gut microbiota, whereby probiotic treatment efficiently reversed the anxiolytic effect, which was comparable to the effect of vagotomy [7, 8]. Mechanistically, the vagal and pelvic nerves control gut’s motility, permeability, hormones secretion, and immune function. This neuronal communication can also sense local host-microbiota interactions in the gastrointestinal tract, and thereby signal the CNS via ENS and sympathetic prevertebral ganglia [9, 10].

### Neuroendocrine-hypothalamic-pituitary-adrenal axis pathway

The hypothalamic-pituitary-adrenal (HPA) axis is the principal neuroendocrine component of stress response [11]. Corticosterone-releasing factor (CRF) is secreted and released from paraventricular neurons of the hypothalamus in response to stress, which then induces the release of adrenocorticotropic hormone (ACTH) from the anterior pituitary. ACTH will stimulate glucocorticoids, mineralocorticoids, and catecholamines from the adrenal cortex, chemicals with multifaceted effects on behavior. For example, glucocorticoids signal to the brain via sensitive receptors throughout the CNS to form an autoregulatory feedback loop. The HPA axis along with its neurotransmitter counterpart, the SNS, produces a series of neural, immunological, and humoral alterations to prime the body for the “fight or flight” response to stress.

In reaction to stress, the HPA axis regulates the release of glucocorticoids, mineralocorticoids, or catecholamines to modulate the intestinal microenvironment [2]. This determines the composition of gut microbiota, intestinal barrier function, and immune and neuroendocrine response. Significant changes in the composition of gut microbiota have been detected in an animal model with early stress including maternal separation and social stress. For example, Wistar rats with neonatal maternal separation (MS) exhibited a significant decrease of anaerobes and clostridia compared with the adult controls without stress. Male CD-1 mice exposed to social disruption (SDR) can reduce the quantity of *Bacteroides* at the cecum and increase the number of *Clostridium*. In circulation, stress has also reduced bacterial genera including *Coprococcus*, *Pseudobutyrivibrio*, and *Dorea*, with an inverse correlation with levels of interleukin (IL)-6 and monocyte chemoattractant protein (MCP-1). In contrast, antibiotic-treated mice exposed to SDR failed to induce an increase of IL-6 and MCP-1 in circulation [12, 13].

Gut microbiota, microbial antigenic cargo, and food are all important HPA axis modulators, which play an indispensable role in neuroendocrine maturation and response. Studies in germ-free (GF) animals underscore a critical role of gut microbiota in the regulation of the set point for HPA activity and behavior response to stress. In contrast to SPF mice, mild restraint stress induced a greater release of corticosterone and ACTH but with a lower degree of anxiety in GF mice. The exaggerated stress response was partially ameliorated by fecal microbial transplant in GF mice and was completely reversed over time by monotherapy of *Bifidobacterium* infants [12]. The reversibility of the exaggerated stress response by microbial colonization is only apparent in mice 9 weeks of age but not in those of 17 weeks of age, which indicates a critical time window in early life for the establishment of neural regulation by gut microbiota [12]. Gut microbiota can modulate the expression of the CRF in the hypothalamus. It can also modulate the expression of brain-derived neurotrophic factor (BDNF), 2A subtype of *N*-methyl-d-aspartic acid receptor (NMDA receptor), and 5-HT1a receptors in the cortex and hippocampus to regulate the functions of HPA axis [13, 14]. Use of probiotics and/or antibiotics, which results in an alternation in the microbiota, drastically changes the region-dependent expression of GABA and BDNF in the brain, and resultant stress-related visceral hypersensitivity and behavior [2]. The impact of microbiota on the HPA seems to be gender-dependent as those alternations were only observed in male mice [15]. In addition to the stress response, the gut microbiota also modulates the limbic system via serotonin and related metabolites [15].

### Immunological pathways

The development, maturation, and function of the mucosal immune system are extensively dependent on microbiota, underlying a potential role of the mucosal immune system in the regulation of emotion and behavior [2]. Segmented filamentous bacteria (SFB) are potent stimuli for the full function of B and T lymphocytes in the gut [16, 17]. As a concept of proof, germ-free (GF) mice lack a functional immune system and colonization with gut microbiota restores their immune function [18]. Gut microbiota communicate with the host through Toll-like receptors (TLRs) [19]. TLR1–10 are commonly expressed in human intestinal epithelial cells, macrophages, dendritic cells, mast cells, lymphocyte, neutrophils, CNS glial cells, and neurons. TLR1–10 can be activated by microbial components, therefore triggering the release of IL-1b, IL-6, IL-8, and TNF-α [19–21]. TLR knockout or transgenic animal models provide strong evidence for the interaction between gut microbiota and immune response via TLR system. For example, TLR2 knockout mice demonstrated gut dysbiosis and aberrant immune responses, which were essential for *Bacteroides fragilis*-mediated prevention of DSS-induced colitis [22, 23]. A study on TLR4 knockout mice suggests that TLR4 mediated Gulf War illness model-induced neuroinflammation and gastrointestinal disturbances via gut dysbiosis and leaky. Results from transgenic villin TLR4 mice suggest that TLR4 can modulate the susceptibility of DSS-induced colitis, which can be transmissible by gut microbiota [24, 25]. In IBD patients, non-synonymous variants in the TLR1, TLR-2, TLR-6, and TLR-9 genes were identified in correlation with impaired host-commensal interaction and distinct disease phenotype [21]. Moreover, the microbiota can also modulate hormonal peptide signaling by synthesis of peptide-like antigenic proteins derived from gut bacteria [2].

### Neurotransmitters, neuropeptides, and microbial-derived metabolic products

Neurotransmitters and neuropeptides are essential regulators for both internal connections within the nervous system and external connections with endocrine and immune system [26, 27]. Many neuropeptides such as substance P, calcitonin gene-related peptide, neuropeptide Y (NPY), vasoactive intestinal polypeptide (VIP), somatostatin, and CRF can modulate the activity of gut microbiota and therefore become important mediators of GBMAx [26]. Conversely, gut microbiota can synthesize and generate a variety of neurotransmitters, neuropeptides, or their precursors, including serotonin, melatonin, histamine, acetylcholine, gamma amino acid, γ-aminobutyric acid, butyric acid, 5-HT, and dopamine. Some of the metabolic products of gut microbiota are an important resource of neural activation molecules. Gut microbiota-derived metabolites from tryptophan metabolism and downstream serotonin, kynurenic, and quinolinic acids are capable of modulating brain function and behavior [28, 29]. Bacterial fermentation products short-chain fatty acids (SCFA) are critical for brain development and CNS homeostasis. SCFA are required for several key neurophysiological processes including microglia maturation, ANS stimulation by enteric neurons, permeability regulation of the blood-brain barrier, and mucosal serotonin secretion [30, 31]. In contrast to molecules activators, d-lactic acid and ammonia generated by bacterial enzymes are neurotoxic products [32, 33].

### Intestinal microenvironment and the blood-brain barrier

The intestinal microenvironment in particular the intestinal barrier and gut microbiota are important modulators of the function of the blood-brain barrier (BBB). The regulatory role of gut microbiota on the function of BBB is supported by experimental evidences from GF mice. Delayed maturation and a persistent permeability defect of BBB were revealed in pregnant GF mice and are associated with reduced protein expression and disorganized tight junction (TJ) [34]. This permeability defect can be restored by FMT from control mice, bacteria strains producing only butyrate or acetate/probionate, or butyrate alone [34]. Gut microbiota can regulate the BBB’s integrity, transportation and secretion of neuroinflammatory substances via several mechanisms: (1) translocating through the disrupted intestinal barrier and interacting with various immune cells, (2) stimulating T cell differentiation and brain infiltration by microbial products, (3) inducing peripheral release of inflammatory cytokines via circulating microbial products (LPS), and (4) directly modulating BBB TJ and glial cells by microbial metabolites (SCFA, tryptophan metabolites) crossing the BBB [34–36].

## Roles of GBMAx in IBD

### Top-down: psychophysiological vulnerability and stress

Preclinical data from animal models reveal that stress is involved in the initiation and relapse of experimental colitis [37]. It has been suggested that stress-induced alterations of GBMAx may exert a deleterious effect on IBD via (1) increasing intestinal permeability and bacterial translocation; (2) changing gut microbiota growth, structure, colonization pattern, and infectious susceptibility to intestinal pathogens; and (3) altering both the mucosal immunity and HPA axis response.

Psychophysiological vulnerability and stress play an important role in the pathophysiology and the course of IBD. Patients have higher rates of diminished psychological functioning and well-being and an increase in panic, generalized anxiety, obsessive-compulsive disorders, major depression, higher distress levels, and stress exposure [37]. In a clinical survey by Pellissier et al., a state of psychological vulnerability has been detected in one half of IBD patients [38]. Some can even precede the initial diagnosis of IBD. The disease progression is considered by the majority of studies as a key driving force for poor psychological outcomes, which further exacerbates chronic health conditions, leading to a lower quality of life (QOL) and higher costs of health care [37, 39]. Furthermore, IBD patients with psychological disorders are associated with earlier diagnosis and onset of IBD. They manifest reduced adherence to treatment recommendations, higher risk of relapse, higher tendency of remission failure with infliximab treatment, and require earlier therapeutic re-initiation [2]. Conversely, improvement of IBD promotes psychological amelioration, which was associated with a better gut and general health, increased activity engagement and symptom tolerance, less pain and perceived stress, and declined medical visits [40]. In clinical practice, antidepressant treatment of concomitant mood disorders in IBD patients exhibits a beneficial effect by decreasing relapse rates and reducing the need for corticosteroids and endoscopies [41, 42].

Clinical outcomes suggest an interaction between IBD and psychological disorders, which is modulated by GBMAx via top-down manner. Neural response and brain imaging research reveal disturbances of emotion circuits and sensory processing in IBD patients separate from that of patients with irritable bowel syndrome (IBS) [37]. In IBD patients, the HPA axis is uncoupled from the SNS, which leads to hypoactive HPA functions after a psychosocial stress and sympathovagal imbalance [2]. In contrast, depression and anxiety suppress the functions of the immune system, therefore triggering an autonomous imbalance of parasympathetic function and sympathetic drive. This imbalance leads to HPA hyperactivity and increased levels of ACTH, cortisol, and CRF in cerebrospinal fluid [37]. Those alternations may explain why IBD may occur following an episode of depression, as stress can cause a profound change in the intestinal immune system. It has been observed that stress induces LPS-stimulated cytokines, leukocyte and natural killer infiltration, platelet activation, and reactive oxygen metabolites with reduced mucosal blood flow in rectal mucosa of patients with ulcerative colitis (UC) [43]. Moreover, stress may generate changes in the non-inflamed areas which are innervated with intact sympathetic nerve fibers and exacerbate inflammatory lesions in Crohn’s disease (CD) [44]. Moderate stressors could affect microbial colonization via modulation on human salivary mucosal secretory glands [45].

### Bottom-up: the gut microbiota

Gut microbiota exert an important impact on the pathogenesis of IBD. An expansion of potential pathogens (*Proteobacteria* phylum, such as *Enterobacteriaceae* including *Escherichia coli*) and global changes in microbial composition (reduced *Firmicutes* species—specifically *Faecailbacterium prausnitzii*) have been described in IBD patients [2]. IBD-associated dysbiosis seems to precede the clinical onset of IBD and is independent of any environmental factors, genetic factors, or even as outcomes from chronic inflammation or medical therapy [2]. However, strong evidence implicating the exact species in IBD patients is lacking [46]. In addition to composition, the metabolism of the microbiota is also profoundly altered in IBD patients. The metabolic pathways of amino acid biosynthesis, carbohydrate metabolism, oxidative stress, and bile salt metabolism have been found altered in the microbiota of IBD patients, strongly suggesting a functional impact of gut microbiota on IBD [2]. Based on all the relevant data, it is generally accepted that the relationship between gut microbiota and IBD is a complex and dynamic interaction rather than causation [47–49].

In IBD patients, there exist an aberrant immune response to microbial dysbiosis due to genetic defects in innate immunity, intestinal barrier, microbial recognition, processing, and phagocyte including nucleotide-binding oligomerization domain-containing-2 (NOD2), Caspase-recruitment domain 15 (CARD15), immunity-related GTPase M (IRGM), autophagy-related 16-like 1 (ATG16L1), and Toll-like receptor (TLR) [50]. The resulting impairment of microbial clearance will persistently stimulate proinflammatory Th1/Th17 polarization and macrophage/monocyte infiltration in the gut, which plays an important role in the immunopathology of IBD [51–53].

Several more recent studies present an excellent example for the modulation by gut microbiota through GBMAx via bottom-up manner in IBD-like colitis and IBD-related neurological complications. In those studies, probiotics can alleviate or prevent memory impairment and anxiety-like behavior in animal models of TNBS or DSS inducing colitis, by increasing BDNF expression and inhibiting NF-κB activation in the hippocampus via restoring gut microbiota disturbances [54–56].

### Targeting GBMAx in IBD via cholinergic modulation

One important GBMAx-mediated therapeutic for IBD is stimulation of the cholinergic anti-inflammatory pathway, either pharmacologically, neurologically, or nutritionally. CNI-1493 is a tetravalent guanylhydrazone that acts as a TNF inhibitor during endotoxemia through the vagus nerve (VN) [57, 58]. In clinical trial, a 12-day treatment with CNI-1493 (8 or 25 mg/m^2^) in CD patients achieved a significant clinical response and a remission rate both at week 4 (67%, 25%) and week 8 (58%, 42%), also with an obvious endoscopic improvement [59]. Galantamine (a central inhibitor for acetylcholinesterase and an allosteric stimulator for nicotinic receptors) and GTS-21 (an α7 nicotinic acetylcholine receptor agonist) also exhibit a cholinergic anti-inflammatory effect and considered a promising therapeutic option for IBD [60, 61]. Encenicline, an α7 nicotinic acetylcholine receptor partial agonist, has recently been reported to alleviate trinitrobenzenesulfonic acid (TNBS)- and dextran sulfate sodium (DSS)-induced colitis [62]. Another encouraging result comes from a study using an animal model of TNBS-induced colitis that a 5-day treatment of VN stimulation performed 3 h per day could effectively improve colitis [63]. Furthermore, high-fat enteral nutrition has also exhibited a therapeutic potential in IBD through releasing cholecystokinin (CCK) and stimulation of vagal afferents [64].

### Microbiota-modulating therapy

Gut microbiota represent another promising therapeutic target of GBMAx for IBD. The microbiota-modulating intervention with clinical potential for IBD patients includes antibiotics, probiotics, enteral nutritional therapy (ENT), and fecal microbiota transplantation (FMT). The significant efficiency of antibiotics exhibited in various animal models of colitis appears to be limited in clinical practice with inconsistent outcomes from a variety of studies [2]. Similar phenomena occurred in the application of probiotics. Although probiotics exhibit some beneficial effect on the treatment of UC and prevention of UC related pouchitis, the efficiency of probiotics on IBD patients remains inconclusive [65, 66].

ENT has been recommended as a first-line therapy for inducing remission in CD with clinical improvement and mucosal healing, especially for pediatric patients [67, 68]. The alternating composition of gut microbiota and a corresponding reduction in lumina antigens and inducing the secretion of anti-inflammatory SCFAs with downstream alterations in T-regulatory cells in the lamina propria was postulated as a possible mechanism [2]. FMT appears to be the most promising microbiota-modulating therapy for IBD in clinical practice. It exhibits a beneficial effect on inducing clinical and endoscopic remission in UC adults based on several lines of evidence derived from double-blind randomized control trials [2]. For the treatment of CD, FMT demonstrated a clinical benefit in pediatric patients in a small cohort study, and high rates of clinical remission and clinical improvement in adult refractory CD in a pilot study [69, 70]. However, clinical challenges and questions remain regarding the safety, durability, procedure standardization, and selection for both donors and recipients.

## Ischemic stroke in IBD

Inflammatory bowel disease (IBD) patients carry a higher lifetime risk (1.5–3.5-fold) for thromboembolism (TE) than in patients without IBD, occurring at a relatively younger age and a higher recurrence rate [71]. Arterial thromboembolism and venous thromboembolism are currently regarded as important extraintestinal complications in IBD patients with considerable morbidity and mortality rates (the overall mortality is 25% per episode) [71]. However, this specific feature of IBD has always been underestimated in clinical practice with only a minority receiving thromboprophylaxis when discharged from hospital [72]. A retrospective monocentric cohort study verified the association between disease activity and the frequency of TE in IBD patients [72]. Therapeutic agents for IBD patients may also represent an impact on the risk of TE. In a cohort study on hospitalized IBD patients, TNF-α inhibitor therapy reduced the risk of TE whereas systemic corticosteroid use was identified to increase the risk of TE [73]. The mechanisms for increased risk of TE in IBD patients have not been completely established. Increasing arterial stiffness, homocysteine and insulin resistance, adipokines produced by the hypertrophic mesenteric fat may all contribute to inflammation-associated atherosclerosis and corresponding increased risk for TE in IBD patients [2]. It is worth noting that arterial stiffness may be alleviated by the treatment of salicylates but not in those treated with steroids and azathioprine or anti-TNF-alpha [74].

Cerebrovascular thromboembolism represented the most frequent and severe central nervous system (CNS) complications of IBD. A population-based retrospective cohort study exhibited a tendency for increased risk for ischemic stroke in IBD patients. The hazard ratio (HR) of ischemic stroke was 1.12 (95% CI 1.02–1.23) among the IBD group versus the non-IBD group [75]. The stratified HR of ischemic stroke was 1.15 (95% CI 1.04–1.28) in CD patients and 1.01 (95% CI 0.84–1.21) in UC patients. The frequency of IBD exacerbation and hospitalization are considered to be risk factors for ischemic stroke. The adjusted HR shifted from 1.07 to 6.36 among the CD patients and from 1.11 to 2.10 among the UC patients with an increasing number of medical visits. Current therapeutic agents aiming at IBD remission seem to modify the risk of cardiovascular or cerebrovascular events [76]. A beneficial effect with increased carotid-femoral pulse wave velocity (PWV) was exhibited with salicylates, but not steroids or azathioprine. TNF-α inhibitors appeared to decrease the risk of ischemic heart disease yet increase the rate of cerebrovascular events. In a nationwide, population-based cohort study from Denmark, the risk of cerebrovascular accidents associated with TNF-α antagonists was 1.42 (95% CI 0.82–2.45). Meanwhile, TNF-α antagonists seem to be a potential risk for ischemic heart disease although no statistical significance was reached [77]. A retrospective study described the clinical characteristics of ischemic stroke in three patients with a history of IBD [78]. Each patient had posterior strokes on at least two separate occasions and/or admitted to the hospital with new strokes at least three times. The link between IBD and posterior strokes is therefore strongly suggested, and factor VIII is identified as a hypercoagulable biomarker associated with increased risk for an ischemic stroke.

## Targeting GBMAx in ischemic stroke

### Top-down: autonomic nervous system

Alternation in intestinal microenvironment is an important pathophysiological consequence of acute ischemic stroke with direct evidence from both experimental models and clinical data. Those changes in MCAO mice include (1) increased gut permeability, (2) impaired gut motility, (3) gut dysbiosis (4) necrosis and shedding of the intestinal epithelium, (5) enteric neuronal loss, and (6) changes in T and B cells in Peyer’s patches (PPs) [79–83]. In patients with acute ischemic stroke, lipopolysaccharide-binding protein (LBP) was associated with both systemic inflammation and a predictive risk of post-stroke infections, which indicates a dysfunction in the intestinal barrier [84]. A brain-to-gut modulation of GBMAx via top-down manner in ischemic stroke has been suggested, as treatment with propranolol or metoprolol (β-adrenergic receptors inhibitors) significantly restored both the gut permeability, and previous pathological changes of caecal microbiota that were mediated by local noradrenaline (NE) release from sympathetic nerves in stroke mice [79, 81].

### Bottom-up: gut microbiota

A significant change in gut microbiota has been detected in stroke mice, which is correlated with stroke outcome. Several potential causative factors are suggested to simultaneously account for the change of gut microbiota after stroke: (1) the suppression of systemic immunity, (2) pro-inflammatory factors released from brain infarction, (3) activation of the SNS, (4) stress induction, and/or (5) impaired intestinal barrier and motility [79, 81]. As determined by next-generation sequencing, Singh et al. identified reduced species diversity and overgrowth of bacteroidetes as a key feature of post-stroke dysbiosis in stroke mice [79]. In a study by Houlden et al., the analysis using 16S rRNA gene amplification followed by pyrosequencing has identified specific shifts in *Peptococcaceae* (increased) and *Prevotellaceace* (decreased), which correlated with both injury severity and neurological deficit [81]. Benakis et al. also suggested several bacterial families including *Verrucomicrobiaceae*, *Prevotellaceae*, and *Clostridiaceae* could be utilized as biomarkers that are capable of predicting infarct volume based on data of family-level phylogenetic classification by fecal 16S rDNA gene frequencies [85].

Experimental models with microbial manipulation including GF animals, antibiotics, and FMT provide more compelling evidences on the correlation between gut microbiota and stroke outcome. Benakis et al. demonstrated that antibiotic (amoxicillin and clavulanic acid)-induced microbial dysbiosis significantly reduced ischemic brain injury in mice after MCAO [85]. This neuroprotective effect was transmissible by fecal transplants from antibiotic-treated mice. In another mouse model of experimental stroke, the outcome was significantly worse after artificially depleting gut microbiota with broad-spectrum antibiotics [86]. Singh et al. recolonize GF mice with post-stroke microbiota and found larger infarction volume and worsen neurological deficits after inducing experimental stroke when compared with GF mice recolonization normal microbiota. In contrast, brain lesion-induced dysbiosis was normalized by therapeutic FMT, with improved stroke outcomes [80]. Clinical data in support of this derives is that alterations in gut microbiota correlate with systemic inflammatory markers (e.g., IL-6, CRP) following stroke [87].

A functional link of gut microbiota, intestinal immune response with ischemic neuroinflammation was strongly suggested by recent investigations, which reflecting a gut-to-brain modulation of GBMAx via bottom-up manner. A microbiota-IL-17-positive T cell-brain axis has been identified central for an explanation of this gut-to-brain modulation in ischemic stroke. Post-stroke dysbiotic microbiota can activate both intestinal innate and adaptive immune response via increasing proinflammatory T-helper cells (Th) Th1 and Th17 polarization and monocyte infiltration [80]. Conversely, microbiota shifts induced by antibiotic (amoxicillin and clavulanic acid or vancomycin) treatment stimulate the regulatory T cells with neuroprotective functions in the gut, which subsequently results in the suppression of pro-inflammatory IL-17-positive γ δT cells mediated by IL-10 [85]. Using in vivo cell-tracking techniques such as fluorescent labeling microinjection and photoconversion in mice, a novel mechanism of intestinal T cells and monocyte trafficking from the gut to the brain in experimental stroke model was observed. The migration of harmful T cells may localize in the leptomeninges and enhance stroke-related neuroinflammation by increasing chemokine production and local infiltration of cytotoxic immune cells [80–85].

Gut microbiota may also play an essential role in post-stroke complications including infection, cognitive impairment, depression, sarcopenia, and weight loss. Stanley et al. identified a translocation and dissemination of commensal bacteria from host gut microbiota in post-stroke infection supported by both clinical and preclinical evidence [79]. Neuronal injury and cognitive deficit in diabetic mice with ischemic brain injury can be alleviated by the supplement of probiotics [88]. Since microbiota shifts occur concurrently with weight changes, cachexia, protein breakdown in skeletal muscle, and mood disorders under other conditions, it is reasonable to speculate a causative role of gut microbiota in post-stroke depression, sarcopenia, and weight loss.

### Alternative therapeutic strategies targeting GBMAx in ischemic stroke

There are limited data available for microbiota-base therapy directly on ischemic stroke. Supplementation with *Clostridium butyricum* exhibited beneficial effects by decreasing neuronal injury and improving cognitive function in diabetic mice with an ischemic brain injury after a bilateral common carotid artery occlusion [89]. Recolonization with normal sham-control gut microbiota or antibiotic-treated (amoxicillin and clavulanic acid) gut microbiota by FMT reduced injury and improved stroke outcome after experimental stroke by MCAO in mice [85]. Furthermore, modulation of gut microbiota by probiotics or prebiotic supplementation of dietary fiber may influence the brain through GBMAx via fortifying the intestinal barrier, regulating microglial activity or augmenting nutrition metabolism of docosahexaenoic acid (DHA) [90, 91]. Therefore, they are expected to provide potential therapeutic implications with significant leverage on ischemic stroke.

Vagus nerve stimulation (VNS) exerts neuroprotective effects through GBMAx via (1) attenuating endotoxemia induced inflammation, (2) decreasing intestinal permeability, and (3) improving the integrity of the blood-brain barrier. Preclinical data demonstrated that VNS could provide both prophylactic and therapeutic protection from traumatic brain injury [2]. It has also been demonstrated to improve motor and cognitive function and also reduce secondary neuronal damage following head injuries [92, 93]. It appears promising to be implicated as a therapeutic tool for ischemic stroke although further investigations are warranted.

Gut-derived neuropeptides offer another GBMAx target. Ghrelin, also known as lenomorelin (INN), is an orexigenic gut hormone with multiple functions including acting as a neuropeptide on modulation of GBMAx. In MCAO ghrelin treatment significantly reduced the neurological deficit and limited infarct size with improved 7-day survival [2]. The possible mechanism may involve exerting antiapoptotic and anti-inflammatory properties in CNS through a vagal pathway, protecting adult rat hippocampal neural stem cells from excessive autophagy and/or relieving intestinal dysfunction and reducing systemic immune response [2].

## Conclusions

**Fig. 2:**
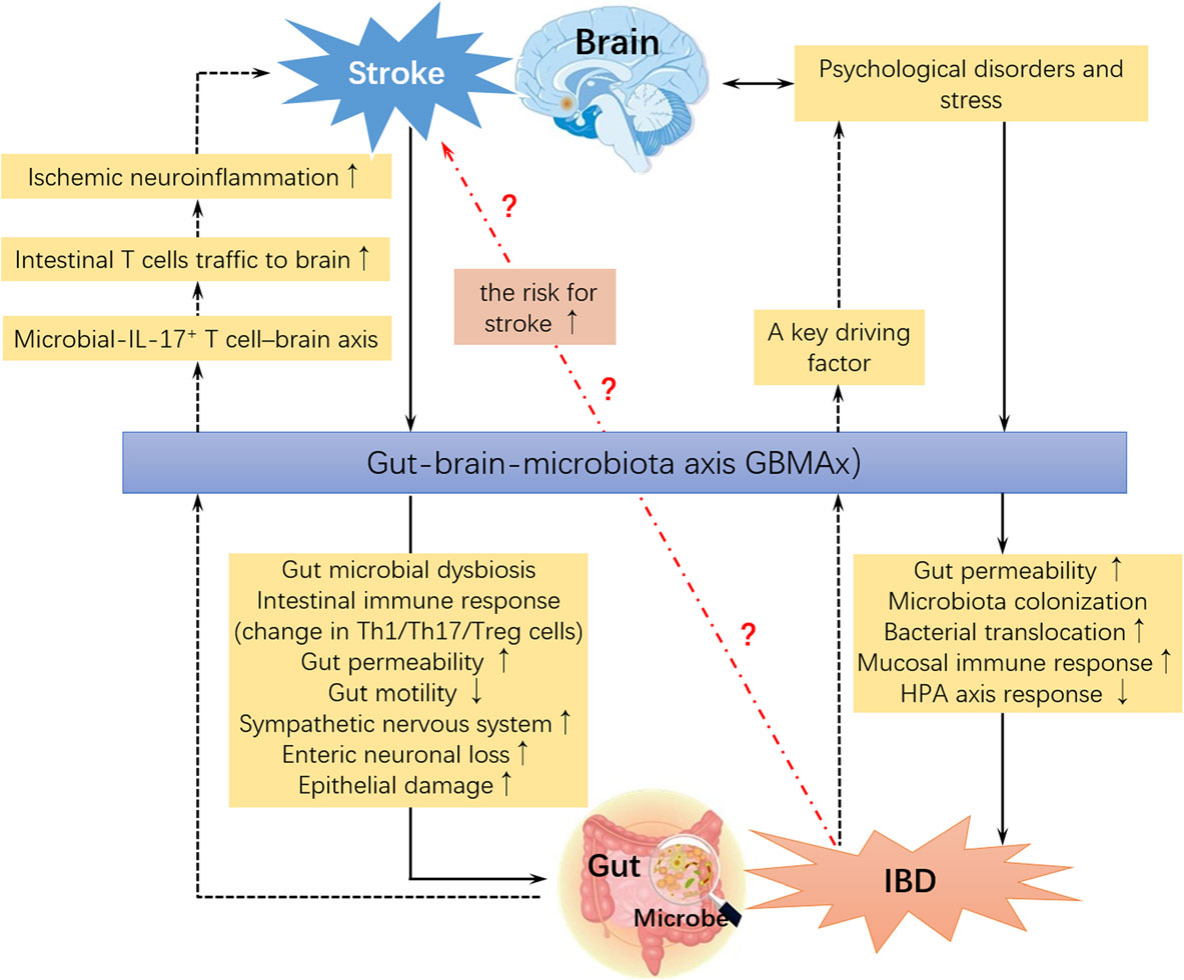
Schematic presentation of the bidirectional interaction of GBMAx in the pathogenesis of ischemic stroke and inflammatory bowel disease (IBD). With ischemic stroke, the excitability of the sympathetic nervous system, enteric neuronal loss, gut permeability, and epithelial damage increases, while gut motility decreases. Gut microbial dysbiosis and the intestinal immune response emerge simultaneously. The changes above are modulated by the GBMAx, aggravating ischemic stroke via microbial interleukin (IL)-17-positive T cell-mediated neuroinflammation. Inflammatory bowel disease (IBD) is a key driving factor for psychological disorders and stress, increasing gut permeability, bacterial translocation, and mucosal immune response and modulating the hypothalamic-pituitary axis response through the GBMAx

An outline summarizing the hypothesis of bidirectional interaction of GBMAx in the pathological mechanism of ischemic stroke and IBD is presented in Fig. 2. Since IBD patients carry higher risks for ischemic stroke, it is highly plausible that GBMAx presents a potential functional link between IBD and increased risk of ischemic stroke. However, studies regarding the role of GBMAx in the relationship between ischemic stroke and IBD are not currently available. The impact of routine therapeutic agents for IBD on the risk and outcome of ischemic stroke remains inconclusive. Recent studies have identified several important components of GBMAx including gut microbiota, proinflammatory T-helper cells (Th) Th1 and Th17 polarization, and macrophage/monocyte infiltration as important mediators in the pathogenesis of both IBD and ischemic stroke, emphasizing its relevance as promising therapeutic targets for stroke, IBD, and stoke in IBD patients. Further research is warranted on the potential role and precise mechanism of GBMAx on ischemic stroke in the context of IBD. It will not only be instructive for accomplishing a better explanation on the higher risk and recurrence tendency of ischemic stroke but also critically necessary to advance promising preclinical trials for novel therapeutics in prevention and treatment of stroke in IBD patients.

## Abbreviations

ACTH: Adrenocorticotrophic hormone
ANS: Autonomic nervous system
BDNF: Brain-derived neurotrophic factor
CD: Crohn’s disease
CNS: Central nervous system
CRF: Corticosterone-releasing factor
DHA: Docosahexaenoic acid
DSS: Dextran sulfate sodium
ENS: Enteric nervous system
GABA: γ-Aminobutyric acid
GBMAx: Gut-brain-microbiota axis
HPA: Hypothalamic-pituitary-adrenal axis
IBD: Inflammatory bowel disease
LBP: Lipopolysaccharide-binding protein
LPS: Lipopolysaccharide
MCAO: Middle cerebral artery occlusion
MCP-1: Monocyte chemoattractant protein, 1
NPY: Neuropeptide Y
PWV: Carotid-femoral pulse wave velocity
QOL: Quality of life
SCFA: Short-chain fatty acids
SFB: Segmented filamentous bacterium
TE: Thromboembolism
TLR: Toll-like receptors
TNBS: Trinitrobenzenesulfonic acid
UC: Ulcerative colitis
VIP: Vasoactive intestinal polypeptide
VN: Vagus nerve
VNS: Vagus nerve stimulation
